# The Role of Spirituality in Pain Experiences among Adults with Cancer: An Explanatory Sequential Mixed Methods Study

**DOI:** 10.21203/rs.3.rs-3425339/v1

**Published:** 2023-10-16

**Authors:** Megan Miller, Stephanie Speicher, Katie Hardie, Roger Brown, William E. Rosa

**Affiliations:** University of Wisconsin–Madison; University of Wisconsin–Madison; University of Wisconsin–Madison; University of Wisconsin–Madison; Memorial Sloan Kettering Cancer Center

**Keywords:** Pain, spirituality, cancer, oncology, mixed methods

## Abstract

**Purpose:**

Foundational research demonstrates that spirituality may affect the way people with cancer experience pain. One potential route is through alterations in thoughts and beliefs, such as pain-related catastrophizing. The purpose of this study is to understand whether spirituality impacts pain experiences through pain-related catastrophizing.

**Methods:**

This explanatory sequential mixed methods study was informed by an adapted Theory of Unpleasant Symptoms. Data were collected via online surveys (N = 79) and follow-up qualitative interviews (N = 25). Phase 1 employed Empirical Bayesian analysis. Phase 2 used deductive content analysis. Phase 3 involved creating a mixed methods joint display to integrate findings and draw meta inferences.

**Results:**

Results indicate that spirituality was directly negatively associated with pain-related catastrophizing, and indirectly negatively associated with the outcomes of pain interference, pain severity, and pain-related distress. Qualitative categories highlight the supportive role of spirituality when facing pain, while also shedding light on the limitations of spirituality in the context of some pain (i.e., severe, neuropathic, and/or chronic). Mixed methods findings reveal the importance of spirituality for some people as they face cancer and cancer-related pain, as well as the need for integrating spirituality as part of a larger pain management plan.

**Conclusions:**

This research advances supportive cancer care by exploring the complex role of spirituality in pain experiences. Findings will inform further exploration into the role of spirituality in supporting holistic symptom management in the context of cancer, as well as developing and testing interventions to enhance spirituality and address symptom-related suffering.

## Background and Significance

Spirituality may be an important source of support when patients are faced with a life-threatening illness such as cancer [7]. “Spirituality” is a human process of connection and meaning-making with self, others, nature, and/or a higher power [41, 43] and may be expressed within or beyond an organized religion. Evidence associates higher self-reported spirituality with positive physical and psychological health outcomes [6, 10]. Prior research suggests that spirituality offers a route to supportive beliefs, thoughts, and emotions, such as a sense of safety, comfort, peace, and tranquility when facing difficult circumstances such as cancer-related pain [4, 28].

Pain-related catastrophizing includes negative cognitions and beliefs related to pain (e.g., perceived lack of control, rumination, magnification, helplessness, expectation of negative outcomes) [33] and can determine how patients experience and respond to pain [27]. Data suggests spirituality may lessen catastrophizing among individuals with chronic non-cancer pain [19, 39]. However, to our knowledge this relationship has not been examined among patients with cancer pain. Understanding whether spirituality impacts pain through pain-related catastrophizing is essential for developing supportive interventions that more holistically address pain among people with cancer.

We aimed to understand the role of spirituality and pain-related catastrophizing in experiences of pain (intensity, distress, and interference) among adults with cancer. More specifically, we sought to 1) quantitatively examine relationships between spirituality and pain outcomes while testing the mediating role of pain-related catastrophizing, 2) qualitatively explore how spirituality and pain-related catastrophizing contribute to pain experiences, and 3) integrate and interpret quantitative and qualitative data to draw meta-inferences about the role of spirituality and pain-related catastrophizing.

## Methods

### Design

This explanatory sequential mixed methods study involved a quantitative phase, a qualitative phase, and a mixed methods phase. Phase 1 included a descriptive correlational analysis of quantitative cross-sectional survey data; phase 2 consisted of semi-structured qualitative interviews with deductive content analysis; and mixed methods data interpretation. Mixed methods integration was achieved in three main ways: 1) connecting (using quantitative data to design and sample for the subsequent qualitative phase of the study [8]); 2) creation of a joint display (integrating both quantitative and qualitative data in a final mixed methods interpretation [18]), and engaging in contiguous reporting (presentation of findings within a single report [16]).

### Theoretical Framework

**Figure 1:**
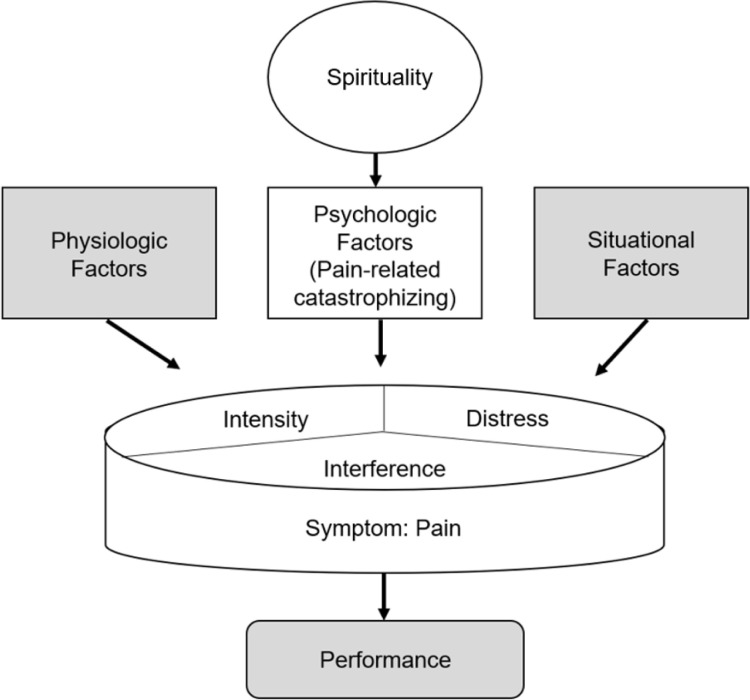
Adapted version of the Theory of Unpleasant Symptoms

An adaptation of the Theory of Unpleasant Symptoms [25] informed this work (Fig. 1). This theory describes the influence of various physiologic, psychologic, and situational factors, the multidimensionality of symptom experiences, and how symptoms influence performance across life domains. Our application focused on how spirituality may impact pain-related catastrophizing (psychologic factor), which may influence experiences of pain (intensity, distress, and interference).

### Participants

Participants were recruited though a local cancer clinic using mailings, phone calls, and in-person recruitment. Eligible participants were age ≥ 18 years, diagnosed with any type or stage of cancer, currently receiving disease-directed cancer treatment, reporting average pain intensity > 0 on a 0–10 scale in the past 7 days, and able to communicate in English. Exclusion criteria included any diagnosed mental illness or cognitive impairment that precluded ethical and safe participation in study procedures.

Phase 1 involved sampling to enroll participants with various levels of pain (minimum 15 people at each pain level [mild = 1–4; moderate = 5–6; severe = 7–10 [38]]). Using the anticipated statistical test (test of joint significance), a sample goal of 80 with 0.8 power allowed for detection of a medium size effect in both a and b analysis paths [17], indicating adequate power. Phase 2 involved sampling a subset of Phase 1 participants to engage in a follow-up qualitative interview.

### Data Collection

In Phase 1, study instruments were primarily completed via Qualtrics. For participants with technology limitations, paper surveys were provided. The following data were collected.

### Demographic/clinical variables.

Standard demographic and clinical characteristics were assessed to characterize the sample, including age, sex, gender, race, ethnicity, education, marital status, comorbidities, type and stage of cancer.

### Pain outcomes.

To assess pain intensity and interference, the Brief Pain Inventory–Short Form (BPI–SF) was used [12]. The BPI–SF measures levels of pain intensity and interference (with general activity, mood, walking, work, relations, sleep, enjoyment of life) using nine self-report items. Pain-related distress was assessed using one item based on the Memorial Symptom Assessment Scale-Short Form, “How much does pain distress or bother you?” on a 0–4 scale where 0 = not at all and 4 = very much [11].

### Spirituality.

Spirituality, operationalized as spiritual well-being, was assessed using the Functional Assessment of Chronic Illness Therapy–Spiritual Well-Being scale [32]. This instrument elicits levels of self-reported spiritual well-being across domains of meaning, peace, and faith with 12 items rated from 0 = not at all to 4 = very much.

### Pain-related Catastrophizing.

Pain-related catastrophizing was assessed using the Pain Catastrophizing Scale [42]. This instrument elicits representations of rumination, magnification, and helplessness, using 13 items on a scale from 0 = not at all, 4 = all of the time.

### Medication use.

A standardized questionnaire was used to elicit information about use of common medications which could influence the experience of pain.

In Phase 2, semi-structured qualitative interviews were conducted. Interview guides were designed to elicit in-depth accounts of participants’ lived experiences with pain, spirituality, and pain-related beliefs and feelings (e.g., catastrophizing). The PI (M.M.) conducted all interviews, primarily through secure Zoom calls, with options for interviewing in-person and via telephone, as needed. Interviews lasted approximately 60 minutes, and were recorded, de-identified, and transcribed.

### Data Analysis

In Phase 1, data analysis was conducted in SPSS version 27 and Mplus Version 8.10. Descriptive statistics were generated for sample demographics and key variables. A test of joint significance [23] was employed to examine 1) the relationship between spiritual well-being and pain-related catastrophizing, and 2) the relationship between pain-related catastrophizing and pain outcomes (intensity, pain-related distress, and pain interference). It was hypothesized that higher spiritual well-being would be associated with lower catastrophizing and that lower pain-related catastrophizing would be associated with lower pain intensity, distress, and interference. Given the relatively small sample size in this study, Empirical Bayesian (EB) [21] was used on a composite indicator structural equation model [26], to address measurement error, with three adjusting covariates (age, sex, and pain medication use). It has been proposed that data-derived informative priors may be obtained from the existing data using EB methods [31]. EB Markov Chain Monte Carlo estimates were used to estimate direct and indirect effects, using data-derived estimates from the dataset as the EB informative priors [31].

In Phase 2, data were analyzed with deductive content analysis using standard procedures and an unconstrained categorization matrix (starting with a priori concepts of interest in the matrix, then allowing additional categories to emerge throughout the analysis process) [14]. An adapted version of the Theory of Unpleasant Symptoms guided identification of concepts and relationships of interest across the study (*spirituality, pain-related catastrophizing, pain outcomes, and relationships between these concepts*) and was used to initially populate the categorization matrix. Engaging with an unconstrained categorization matrix allowed for nuanced findings to emerge from the data, expanding upon the initial matrix. Individual coding team members read each transcript multiple times to ensure a thorough understanding of content. The team then developed the unconstrained categorization matrix and re-examined each transcript line by line, coding data according to categories and subcategories (adding to the matrix as necessary), then organizing findings into meaningful statements exemplified by representative quotes. Maintaining analytic memos, collaborative analysis, and regular consensus-building meetings enhanced trustworthiness and rigor [13].

Phase 3 involved connection, integration and interpretation of mixed methods data through the creation of a novel joint display [8, 18]. The joint display was created to illustrate key quantitative and qualitative findings, and to identify meta-inferences across all findings. In alignment with mixed methods, narrative reporting was employed, with qualitative, quantitative, and mixed findings reported using a contiguous approach [16].

### Ethical Considerations

The University of Wisconsin-Madison Institutional Review Board reviewed and approved study procedures before beginning (protocol #2021 – 0143). A detailed informed consent form was included at the start of the study survey, with options to consent and proceed, or to decline and end the survey. For completion of Phase 1, participants received $10 in compensation via a gift card. Those who completed Phase 2 received an additional $20 in compensation, also via a gift card. A team member was available to answer questions about the study during the consent process. Participants were assured they could end participation at any time with no adverse consequences to care received.

## Results

**Figure 2:**
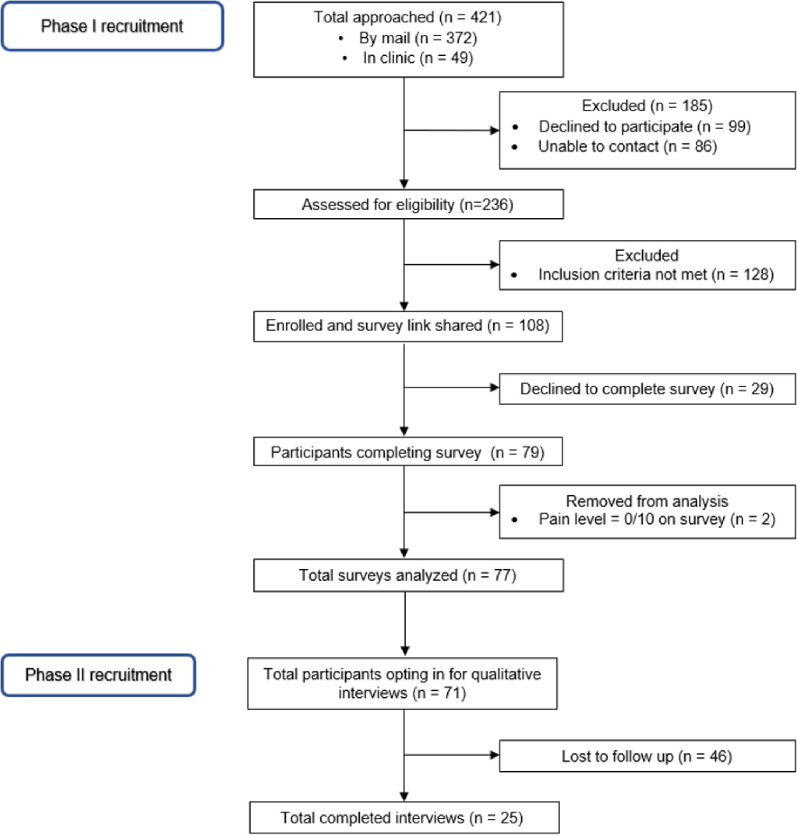
Study flow diagram

Data were included from N = 77 quantitative surveys and N = 25 qualitative interviews. A study flow diagram is presented in Fig. 2. Demographic characteristics of the sample are provided in Table 1.

### Phase 1: Quantitative

EB analysis results (Table 2) indicate that spiritual well-being was directly negatively associated with pain-related catastrophizing (estimate=−1.818, p < 0.036) and was indirectly negatively associated with the three outcomes of pain intensity (estimate=−0.151, p < 0.036), distress (estimate=−0.114, p < 0.036), and interference (estimate=−0.291, p < 0.036). Pain-related catastrophizing completely mediated spiritual well-being to average pain and to pain-related distress, but only partially mediated spiritual well-being to pain interference. The fit of the model was considered appropriate with Comparative Fit Index = 0.982, and Tucker-Lewis Index = 0.948, and a posterior predictive P-value (PP) of 0.443. The PP measures how well the model predicts new data based on observed data. In other words, it measures the probability that new data would have the same value given the observed data and model, thus providing a measure of fit. A PP of 0.443 means that the model predicts that new data would have a 44.3% chance of having the same value as observed data. Overall, the Bayesian structural model appears to provide a good fit to the data based on fit statistics and information criteria.

### Phase 2: Qualitative

Three discrete yet interconnected categories and nine sub-categories were identified in the qualitative analysis. These further clarify how spiritual well-being and pain-related catastrophizing contributed to experiences of pain. Categories and sub-categories are presented below. Table 3 provides representative quotes from participants which serve to expand on each category.

Category 1) Pain-related catastrophizing is a pervasive phenomenon, often marked by: a) Spirals of mental stress and fear related to pain, b) Anger and frustration with pain and its impacts on quality of life, and c) Hopelessness in the face of pain. This category illustrates the complex and pervasive role of pain-related catastrophizing in the lives of many participants who are facing pain. Findings reveal the interwoven, reciprocal experiences of catastrophizing and pain. Participants described their experiences with pain “spirals”, as well as the “mental anguish” they found hard to disentangle from the painful experience itself, bringing enhanced complexity to the understanding of suffering associated with cancer pain.

Category 2) Spirituality can help patients transcend pain-related catastrophizing through: a) Inspiring faith and trust serving as motivation to continue amidst suffering, b) Helping provide meaning and a wider perspective around pain, and c) Offering emotional comfort through supportive beliefs and practices. Spirituality was found to provide a framework for interpreting and making meaning around experiences of pain. Participants described how their spirituality motivated and inspired them to keep caring for themselves despite their suffering. Engaging with spirituality also helped some participants hold a broader perspective around their pain experiences, often leading to intentionally focusing on gratitude amid pain and the ability to hold a “both/and” perspective (e.g., having pain and still feeling grateful that things could be worse). Spiritual beliefs and practices helped participants self-regulate and access feelings of calm when facing pain and the stress that comes with it. Overall, many participants described routes by which spirituality acted as an antidote to pain-related catastrophizing and altered the way they could relate to their pain experiences.

Category 3) Spirituality has real limitations in the context of certain pain experiences, such as when pain is: a) Chronic, b) Neuropathic, and/or c) Especially intense/severe. It is especially relevant to note that some participants communicated the limitations they experienced when exploring how spirituality might impact their pain. For example, various participants were facing neuropathic pain and described how nothing else could be done to help. While spirituality may be supportive in some contexts, limitations must be recognized.

### Phase 3: Mixed Methods

A joint display was created (Table 4) illustrating key findings across Phases 1 and 2 and drawing meta-inferences about the role of spirituality and pain-related catastrophizing in pain experiences. Results focus on spirituality as an important part of the journey with cancer and pain for many participants. Findings also highlight moderate levels of spirituality and a wide range of meanings/lived experiences, moderate levels of pain-related catastrophizing with features of anger, helplessness, fears about the future, and wide-ranging experiences with pain (various causes, descriptions, and management attempts). Findings support that spirituality is useful for some patients when facing pain-related catastrophizing and pain.

## Discussion

This mixed methods study highlights the supportive role of spirituality for some people with cancer pain. Findings align with professional recommendations stating the necessity of incorporating spirituality as part of serious illness care [6, 15]. Quantitative, qualitative, and mixed methods results supported that spirituality is related to pain-related catastrophizing, and to pain experiences, via various possible routes. Within this context, limitations to spirituality’s usefulness for some participants were also uncovered. Findings from this study can inform researchers and clinicians in designing interventions that can improve pain management through increased engagement with spirituality.

Findings that spiritual well-being was directly negatively associated with pain-related catastrophizing, and was indirectly negatively associated with the selected pain outcomes (intensity, pain-related distress, and interference), support hypothesized relationships identified through the theoretical framework [25].

Findings extend previous work on the relationship between spirituality and pain, which have yielded mixed results. For example, a prior secondary analysis of spirituality and pain among women with breast cancer receiving foot reflexology found no significant relationship [30]. However, another secondary analysis among patients with solid tumor cancers undergoing chemotherapy found that spirituality trended toward an association with lower pain severity, although results were not significant (p < .058), and that spiritualty was significantly associated with lower pain-related interference, but not distress [29]. A cross-sectional study of Black patients treated for cancer pain revealed associations between higher spirituality and lower pain severity, pain interference, and total symptom scores [4]. Differences in findings could be due to variations across study designs, diversity in participants among the samples, and/or reflective of real-world differences in the importance of spirituality across contexts. Further clarification is needed to better understand the role of spirituality in symptom experiences, and particularly which patients may or may not benefit from incorporation of spirituality in pain management plans.

While the relationship between spirituality and pain-related catastrophizing has not yet been explored among people with cancer, one recent study reported a significant relationship between faith and positive expectations (i.e., optimism and general self-efficacy) among people with cancer [45]. Given that pain-related catastrophizing includes components of magnification, helplessness, and expectation of negative outcomes [33], interventions focused on enhancing faith, optimism, and self-efficacy could improve pain-related catastrophizing. Future research grounded in the adapted theory [25] which focuses on delineating spirituality and these nuanced “psychologic factors” is warranted.

Prior qualitative studies have shown that spirituality is centrally important to many people when facing cancer [1, 28]. Spirituality can offer a sense of purpose, provide comfort and hope, and bolster determination amid cancer-related suffering [1, 9] Higher spiritual well-being has been related to mental adjustment strategies such as “fighting spirit” [20]. These prior findings align with our results, especially that spirituality can help patients transcend pain-related catastrophizing through inspiring faith and trust, and by motivating a person to continue amid suffering. One recent study found that spirituality did not significantly relate to pain, yet resilience had a direct and negative correlation with pain [3]. In the current study, participants often described spirituality serving as a source of meaning and resilience in the face of suffering. Further, antecedents to pain in the context of cancer have been identified to include meaning ascribed to pain and personal perceptions of pain [22], and a recent meaning-centered intervention yielded significant reductions in pain severity, pain interference, and pain self-efficacy [44].

Mixed methods findings centered on the importance of spirituality for some participants as they faced cancer and cancer-related pain, as well as the need for integrating spirituality as part of a larger pain management plan. Though spirituality can effectively reduce cancer pain [2], research is needed to clarify which participant characteristics, contexts, and/or types of pain might create conditions for spirituality-centered interventions to be optimized. Interestingly, higher levels of pain are associated with greater spiritual distress [35], and effective pain management may improve spiritual well-being and psychosocial outcomes among people with cancer [45], opening possibilities of spirituality and pain existing together in a more complex, reciprocal relationship. Based on these findings, our adapted theoretical framework [25] may be well suited to additional modification to explore this possibility. Future longitudinal research is needed to clarify the nature of relationships between spirituality and pain, and to explore potential mediators and moderators, such as pain-related catastrophizing, optimism, mindfulness, interoception, and neurophysiological changes.

Recent research has revealed associations between engagement with spiritual care and less aggressive care at the end of life, more time between final treatment and death, and increased use of hospice services [37]. These outcomes each can yield improved quality of life and reduced healthcare costs [37]. While this study specifically examined spirituality’s role in pain experiences, it is important to note that the role of spirituality in cancer care is far-reaching and continues to be explored.

Evidence-based spiritual screening tools have been developed for use in practice [34], and the National Coalition for Hospice and Palliative Care recommends in-depth spiritual screening and history at initial patient encounter, with subsequent regular spiritual assessments in tandem with other clinical assessments, especially upon changes in clinical status [15]. Regular documentation and communication about spiritual care needs and preferences is warranted within interdisciplinary teams. Spiritual care may become increasingly important in times of existential distress or clinical uncertainty [6, 7], thus, it is crucial that clinicians familiarize themselves with the roles spirituality plays in the lives of patients and their caregivers. Clinicians must be aware of patients’ spiritual beliefs, and also their practices, to ensure adequate incorporation in clinical settings, including participation of religious and faith community members, health system chaplains, and other resources needed to promote spiritual well-being. A recent in-depth review established evidence-based statements related to spiritual care, including: 1) incorporating spiritual care into care of patients with serious illness, 2) incorporating spiritual care into education and training of interdisciplinary team members who care for persons with serious illness, and 3) including specialty spiritual care practitioners on teams who care for persons with serious illness [6]. Findings of this study align with these recommendations, highlighting the role of spirituality as an aspect of holistic pain management among people with cancer.

Finally, most people with cancer experience more than one symptom [24], supporting a need to assess and address symptoms concurrently. While this study focused exclusively on the symptom of pain, future research to explore spirituality-centered interventions and their role in managing co-occurring symptoms could yield a greater impact. One recent study reported improvements in quality of life, pain, nausea, and vomiting after a 1-month spirituality-based palliative care intervention [36]. Other such interventions that may increase spirituality and positively impact cancer-related symptoms include psilocybin-assisted therapy, direct spiritual care, and life reviews [5, 40], each warranting ongoing investigation.

## Limitations

Despite the rigorous mixed methods approach, several limitations were identified. First, given the nature of cross-sectional data collection, this is an exploration of potential relationships, and is not able to prove causality. Additionally, many participants who had severe pain declined to participate in one or both phases, citing reasons such as being too busy or too sick. While the research team made conscious and earnest efforts to engage with a more diverse group of patients, the sample did not include any gender non-conforming individuals and does not represent the larger population in terms of race and ethnicity. Given that interviews and surveys were conducted in English, findings also do not represent those who speak other languages. The lack of diverse voices in this study, accompanied by the fact the research team only recruited the people most open to discussing their pain and spirituality, could have limited the inclusion of other relevant perspectives. Also, the overall small sample size made quantitative modeling challenging. Therefore, findings are limited in terms of generalizability and translatability. Finally, of participants who completed both the survey and the interview, some informally reported changes in their cancer treatments, pain experiences, and spirituality between Phases 1 and 2, which should be taken into consideration in the context of this mixed methods study.

## Conclusions

Given the need for holistic support in the management of pain among people with cancer, additional exploration into the role of spirituality is warranted. While spirituality can be unique to each person, aspects of connection and meaning can be relevant in the lives of patients across contexts. Findings from this mixed methods study support the idea that consciously engaging with spirituality can serve as a sustaining resource amidst challenges such as cancer, pain-related catastrophizing, and pain.

## Figures and Tables

**Table 1:** Demographic characteristics

Variable		Mean, S.D. (min-max)
**Age**		
		62.7, 12.4 (28–90)
Variable	n	%
**Sex**		
Male	29	37.7
Female	48	62.3
**Gender**		
Man	29	37.7
Woman	48	62.3
Other	0	0
**Race**		
Black or African American	4	5.2
Native American or Native Alaskan	2	2.6
White	69	89.6
Asian or Pacific Islander/Multiracial/Other	2	2.6
**Ethnicity**		
Not Hispanic/Latina	76	98.7
Hispanic Latina	1	1.3
**Employment**		
Full-time	15	19.5
Part-time	3	3.9
Retired and/or unable to work	56	72.7
Not employed and seeking work	1	1.3
Other	1	1.3
Prefer not to answer	1	1.3
**Education**		
High school graduate (or GED) or some high school	12	15.6
Some college or 2-year degree	29	37.7
4-year college graduate	16	20.8
More than 4-year college degree	5	26.0
**Marital status**		
Never married	1	1.3
Widowed	4	5.2
Divorced/Separated	13	16.9
Member of an unmarried couple	9	11.7
Married	50	64.9
**Religious or Spiritual identity**		
Christian	49	63.6
Other	14	18.2
Not applicable	9	11.7
Prefer not to answer	5	6.5
**Cancer Type**		
Breast Cancer	19	24.7
Lung Cancer	17	22.1
Prostate Cancer	3	3.9
Colorectal Cancer	3	3.9
Skin Cancer/Stomach Cancer/Other	22	28.6
Multiple Cancers	13	16.9
**Current Cancer Treatment**		
Chemotherapy	23	29.9
Radiation therapy	0	0
Surgery (within the last month)	0	0
Hormonal therapy	14	18.2
Targeted therapy/Immunotherapy	14	18.2
Multiple types	19	24.7
Unknown/Prefer not to answer	7	9.1

**Note.** *Because of rounding, percentages may not total 100.*

**Table 2:** Phase 1 results

	Estimate	Posterior S.D.	P-Value 1-tailed test	95% CI 1-tailed test
**Direct Effects**				
Spiritual well-being à Pain-related catastrophizing	−1.818	1.018	0.036*	−3.487, −0.158
Spiritual well-being à Pain interference	−0.563	0.341	0.051	−1.129, 0.003
Pain-related catastrophizing à Pain interference	0.166	0.031	< 0.001*	0.116, 0.216
Spiritual well-being à Average pain intensity	0.108	0.242	0.325	−0.291, 0.504
Pain-related catastrophizing à Average pain intensity	0.088	0.020	< 0.001*	0.055, 0.120
Spiritual well-being à Pain-related distress	0.046	0.123	0.370	−0.158, 0.244
Pain-related catastrophizing à Pain-related distress	0.064	0.010	< 0.001*	0.047, 0.081
**Indirect Effects**				
Spiritual well-being à Pain-related catastrophizing à Average pain intensity	−0.151	0.098	0.036*	−0.333, −0.014
Spiritual well-being à Pain-related catastrophizing à Pain-related distress	−0.114	0.069	0.036*	−0.235, −0.011
Spiritual well-being à Pain-related catastrophizing à Pain interference	−0.291	0.181	0.036*	−0.615, −0.027

* =significant at p < .05

**Table 3:** Phase 2 results

Category / Sub-category	Representative Quotes
Pain-related catastrophizing is a pervasive phenomenon, often marked by...	
a. Spirals of mental stress and fear related to pain.	*“The mental stress [of pain] has been unbelievable.”* *“[My doctor] says that he wanted me to be more and more focused on new pain. If my elbow starts to hurt and I haven’t done anything it’s reason to be concerned because that cancer cell could have split and gone to my elbow or the bone marrow there... that is probably the hardest part of the journey... I get into these pain spirals.”* *“It’s, for me, very difficult to separate the pain from mental anguish from physical pain ’cause when I’m in situations where I have a lotta stress, my body hurts. It’s really... all those things that go into what stress does to your body... Even just holding yourself too tight.”*
b. Anger and frustration with pain and its impacts on quality of life.	*“The pain hurts, and it makes you angry. Sometimes it makes you sad. It’s just anger. Anger and fear. Fear comes from the anger, or anger comes from the fear. When is it gonna stop? Or is it ever gonna stop? Or am I gonna die before it stops? You know? I think that the most of all of it is just the fear.”* *“I just don’t have cancer. There’s so much pain affiliated with it. I try not to be a baby about it, but sometimes I just want to say, ‘Listen, people. I’m hurting today. Okay. I just can’t deal. I’m hurting.’”*
c. Hopelessness in the face of pain.	*“Sometimes pain and other things can just be so bad that you just think—there was one point where* I *thought...‘I think this is it for me’.”* *“I know pain, chronic pain especially... knowing that your disease is terminal and thinking you’re gonna live the rest of your life in this amount of pain or more, it’s just gonna get worse and worse, is terrible.”*
Spirituality can help patients transcend pain-related catastrophizing through.	
a. Inspiring faith and trust, serving as a motivation to continue amidst suffering.	*“When you have [pain] or anything like that, try and do whatever it is that makes you feel comfortable, but also realize that it’s not gonna be there forever. You just have to let go of it... let go and let God.”* *“It just takes so much mental toughness to say that I’m in pain, and I’m groggy, and I am backed up and bloated, and I’m still gonna get up, and I’m gonna walk. That I’m gonna walk up and down the hallway three times, this time. That’s the toughness it requires... [Tearfully]... Before I went into the surgery the second time, [my motivation] was family, and it was the number of things I had to live for. Better times... I am quite spiritual... To get up off the deck when every muscle in your body tells you to just lay back and chill... I believe that the spirituality will give you—will help motivate you.”* *“God’s always watching, and He’s always got his hand right there for you to reach out when you need it. That’s how that go, in my opinion.”* *“I’m a big control freak, not of other people but of my own self... This [cancer-related pain] has been an interesting journey into maybe you don’t have to know everything... It’s important to remember that there are things bigger than us in a very nonspecific way. I don’t have a God or a thing that I do to acknowledge that, but reminding myself periodically, ‘There are things that are bigger and smarter and more powerful than I am at play here’... [Laughter] Like, “Okay, well this thing is here. This pain is here... Maybe I need to live with it for a little bit and see what that means for me.”*
b. Helping provide meaning and a wider perspective around pain.	*“There has been a lot of pain, but because I have tried so hard to be positive about everything, that God has kinda given me a gift of not emotionally feeling it as much as I could.”* *“When I’ve been in physical pain, I try not to focus on it. I try to focus on something that is brighter or sunnier... I recognize that it’s there...it hurt[s]... and I move on. I don’t let it be the [only] thing... I don’t let it control my day. I’ve been very fortunate.”* *“I have medical insurance. I have enough money to live until I die. There are so many people who are just struggling for all that. I have physical pain. There’s times where I’m up half the night ’cause I can’t get my legs to stop hurting. In the whole scheme of things, it’s manageable.”* *“Like when I went through the surgery... just to realize that I might be feeling the pain, but there’s other people in this world that feel a lot more pain that are a lot harder—a lot more hurtful than the pain that I’m feeling. I kind of try to turn it around.”*
c. Offering emotional comfort through supportive beliefs and practices.	*“I don’t ‘meditate’ as a daily thing... One thing that I did do, I learned a technique called the release technique, which is basically whatever it is that you’re holding that is inside of you, that is bothering you, that is troubling you, annoying you, learning how to let those things go, release them, let ’em go.... That is the spirituality that I think has helped me deal with all of this stuff, deal with the pain.”* *“Well, if I have a bad night [with pain]... I pray that it’ll go away but I pray His will be done, not mine... I just feel spiritually that He does help me through it... I pray, and I sort of meditate... They are helpful, and like at night, it’s wonderful because I can pray and go to sleep. And, you know, I don’t sleep real well.”* *“I believe that having that spirituality and the belief in that higher power helps me stay calm most of the time, helps me believe that there’s something more after this world, I find comfort in that and that everything somehow is gonna be okay in the end.”* *“I try to get a sense of calm. It’s not as hard as it used to be when I used to get the panicky feeling... Thinking of things that are bigger than me and then just trying to remain calm, get through it, breathe through it, do what you can to make [the pain] better.”*
Spirituality has real limitations in the context of certain pain experiences, such as when pain is...	
a. Chronic.	*“I have neck pain daily and I know spiritually there’s really nothing that can be done because it’s chronic pain, I can pray all I want, but the pain is still gonna be there.”*
b. Neuropathic.	*“I know this [neuropathy] is a crazy side-effect to deal with, I know many, many people deal with it for cancer, for diabetes, for all kinds of things, I know there’s a lot of research being done on what can people do, but nerves are nerves.”*
c. Especially intense/severe.	*“[It was] like 4 out of 10 pain-wise for a while. Now, I cruise at about a 5 or 6 constantly. At night, it can even be up to an 8 or a 9. I gave birth three times, so saying that it’s—and I have an incredible threshold for pain... I just keep hoping that maybe it’ll go away... I can’t expect miracles.”*

**Table 4:** Phase 3 results

Concept	Quantitative Mean, S.D. (min-max) or Estimate/p	Qualitative	Mixed
**Spirituality / Spiritual well-being**	1.89, .64 (.25–3.33 out of 0–4)	Participants described spirituality as connection to self, to something greater (nature, Higher Power, community), and a sense of meaning or purpose. Lived experiences of spirituality were unique, yet often were reported to offer benefits when facing cancer and mortality.	Spirituality levels were moderate in this sample. Meanings of spirituality and the ways it appeared in everyday life varied widely.
**Pain-related catastrophizing**	15.24, 9.25 (0–39 out of 0–52)	Pain-related catastrophizing was described by several participants, with a focus on “spirals” of mental stress and fear, anger and frustration with pain, and feelings of hopelessness.	Pain-related catastrophizing levels were moderate in this sample. Lived experiences with pain-related catastrophizing varied across participants (anger, helplessness, fears about the future).
**Pain**	Worst pain 5.29, 1.99 (1–9 out of 0–10) Average pain 3.70, 1.62 (1–8 out of 0–10) Pain-related distress 2.36, .90 (1–4 out of 0–4) Pain interference 3.79, 2.56 (0–8.86 out of 0–10)	Pain was described as originating from various causes (cancer, cancer treatment, other co-occurring conditions, and unknown origins). Participants described pain as a pervasive and challenging aspect of their lives, and attempted various strategies to manage it (medications, topical treatments, lifestyle modifications, etc.)	All participants in the study reported some level of pain, yet there was a range of experiences with pain across individuals. Average levels were mild and worst levels were moderate. A range of causes, descriptions, and attempts to manage pain were explored.
**Relationship between spiritual well-being and pain-related catastrophizing**	Estimate: −1.818 p = 0.036*	For some participants, spirituality offered a respite from pain-related catastrophizing, providing an opportunity to zoom out, connect with meaning and a wider perspective on life. Faith, trust, and emotional comfort brought about by spirituality could help facilitate transcendence of spirals of pain-related worry and anger.	Findings support the idea that spirituality is helpful for some patients when facing pain-related catastrophizing. Various possible mechanisms underlying this relationship were identified and explored.
**Relationship between spiritual well-being and pain experience**	Average pain Estimate: −0.151 (p = 0.036*) Pain-related distress Estimate: −0.114 (p = 0.036*) Pain interference Estimate: −0.291 (p = 0.036*)	For some participants, spirituality served as a support when facing pain, inspiring motivation to continue with self-care activities amidst suffering, helping provide meaning around pain, and offering comfort/emotional regulation which had the capacity to alter the way pain was experienced. Other participants reported that spirituality did not help them when facing pain.	Findings support the hypothesis that spirituality is helpful for some patients when facing pain, yet limitations of spirituality’s usefulness in the face of pain were also revealed.
